# Antifolate agent aminopterin demonstrates potent anti-monkeypox virus activity *in vitro* and *in vivo*

**DOI:** 10.1128/spectrum.01581-25

**Published:** 2026-06-22

**Authors:** Jing Wu, Meimei Duan, Lin Cheng, Deyan Chen

**Affiliations:** 1 Interdisciplinary Eye Research Institute (EYE-X Institute), Bengbu Medical University 74539 , Bengbu, China; 2 Key Laboratory of Infection and Immunity of Anhui Higher Education Institutes, Bengbu Medical University 74539 , Bengbu, China; 3 School of Public Health, Fujian Medical University 74551, Fuzhou, China; 4 Institute for Hepatology, National Clinical Research Center for Infectious Diseases , Shenzhen Third People's Hospital 638560 , Shenzhen, China; 5 Medical School, Nanjing University 12581, Nanjing, China; Engelhardt Institute of Molecular Biology, Moscow, Russian Federation

**Keywords:** monkeypox, monkeypox virus, antifolate, aminopterin, antiviral

## Abstract

The ongoing spread of the monkeypox outbreak underscores the urgent need for the development of effective therapeutics against the monkeypox virus (MPXV). Aminopterin is a classical antifolate agent known for its anti-tumor activity through the inhibition of folate metabolism. Here, aminopterin was identified as an effective anti-MPXV agent. It significantly inhibited MPXV replication, unlike other antifolate agents such as methotrexate, pemetrexed, and LSN3213218. In Vero E6 cells, the half-maximal inhibitory concentration (IC₅₀) was 36.10 nM, the half-cytotoxic concentration (CC₅₀) was 3,310 nM, and the selectivity index (SI) was 91.690. In human foreskin fibroblast cells, the IC_50_ was 32.73 nM, CC₅₀ exceeded 10 μM, and SI was greater than 305.530. *In vivo*, aminopterin not only inhibited MPXV infection but also mitigated high-dose viral infection-associated inflammation. Furthermore, the data indicated that aminopterin reduced viral load by interfering with viral attachment and replication. Together, these findings identify aminopterin as a promising candidate for anti-MPXV drug development.

**IMPORTANCE:**

The urgent need for effective monkeypox virus (MPXV) therapeutics is underscored by the ongoing outbreak. This study identifies aminopterin as a potent anti‑MPXV agent among four antifolate agents. Crucially, aminopterin suppresses viral infection and mitigates infection-associated inflammation *in vivo*. These findings position aminopterin as a potential candidate for the development of an antiviral agent against MPXV.

## INTRODUCTION

The global monkeypox (Mpox) outbreak has affected more than 90,000 individuals across 117 countries since it emerged in 2022 (1). Monkeypox virus (MPXV), a double-stranded DNA virus, was first isolated from cynomolgus monkeys in Denmark in 1958 (2). Its zoonotic potential was confirmed following the identification of human cases in the Democratic Republic of the Congo in 1970 (3). Mpox infection typically begins with non-specific symptoms such as pain, fever, fatigue, and lymphadenopathy, which are followed by a rash. The rash usually starts on the head and face before progressing to other parts of the body (4, 5). This eruptive phase generally lasts 2–4 weeks (6). Although most cases are self-limiting, Mpox can cause severe diseases in immunocompromised individuals and during large outbreaks, highlighting the urgent need for effective antiviral treatments (7). Therapeutic options for MPXV infection remain limited, with only two antivirals, tecovirimat and brincidofovir, originally United States Food and Drug Administration-approved for smallpox, currently available for clinical use (8). Therefore, the development of targeted anti-MPXV therapeutics is essential to shorten the disease course, alleviate symptoms, and prevent future epidemics.

Aminopterin (4-Aminofolate) is an antifolate agent that demonstrated efficacy in the chemotherapeutic management of childhood acute leukemia in the late 1940s (9). It exerts its primary pharmacological effect by inhibiting dihydrofolate reductase (DHFR), thereby blocking cellular folate utilization (10). In a previous study, we demonstrated the antiviral activity of various antifolate compounds, including methotrexate, aminopterin, pralatrexate, and pemetrexed, against vesicular stomatitis virus, an enveloped negative-sense RNA virus with zoonotic potential (11). However, the anti-MPXV activity of aminopterin remains largely unexplored.

In this study, we evaluated the anti-MPXV activity of four antifolate compounds. Our results demonstrate that aminopterin exhibits significant antiviral activity against MPXV *in vitro*. We further confirmed its efficacy *in vivo* using a well-established MPXV-infected Spanish dormouse (*Dryomys nitedula*; *Rodentia*, *Gliridae*) model. This animal is a natural host of MPXV and has been used for the pharmacological evaluation of MPXV infection (12). These findings provide critical insights for the development of novel anti-MPXV drugs.

## MATERIALS AND METHODS

### Chemical reagents and solutions

Methotrexate (MTX; Cat# M813626) and aminopterin (Cat# A800526) were purchased from Macklin Biochemical Co., Ltd (Shanghai, China). Pemetrexed (Cat# M11536) and LSN3213128 (Cat# HY-107981) were purchased from Molnova (Ann Arbor, United States) and MedChem Express (Shanghai, China), respectively. All compounds were dissolved in dimethyl sulfoxide (DMSO) to prepare a stock solution (10 mM) and stored at −20°C. Cell culture reagents, including fetal bovine serum (FBS; Cat# 10099141), Dulbecco’s Modified Eagle Medium (DMEM; Cat# 21010046), and a bicinchoninic acid (BCA) protein assay kit (Cat# 23225), were purchased from Thermo Fisher Scientific Co., Ltd (Shanghai, China). A Cell Counting Kit-8 (CCK-8; Cat# C0038) was acquired from Beyotime Biotechnology (Shanghai, China). Molecular biology reagents, including ABScript III RT Master Mix (Cat# RK20429) and SYBR Green Fast qPCR Mix (Cat# RK21203), were sourced from ABclonal (Wuhan, China). DNA extraction was performed using the Universal Genomic DNA Kit (Cat# CW2298M) from CoWin Biotechnology (Jiangsu, China).

### Cells, viruses, and animals

Vero E6 cells (African green monkey kidney epithelial cells) were provided by Fujian Medical University (Fuzhou, China), while the human foreskin fibroblast (HFF) cell line was purchased from Hunan Fenghui Biotechnology (Hunan, China). Both cell lines were maintained in DMEM supplemented with 10% heat-inactivated FBS and 1% penicillin–streptomycin at 37°C in a humidified incubator with 5% CO_2_.

The MPXV strain SZTH42 (isolated from a 37-year-old male patient in June 2023) was provided by Shenzhen Third People’s Hospital (13, 14). MPXV experiments were conducted under Biosafety Level 3 (BSL-3) conditions using Vero E6 cells. The virus was propagated in Vero E6 cells. Viral stocks were diluted at a 1:5 ratio with culture medium consisting of DMEM supplemented with 2% FBS and 1% antibiotics (penicillin 5,000 IU/mL and streptomycin 2,500 µg/mL). After 6 h of incubation at 37°C with 5% CO_2_, the supernatant was replaced with fresh medium. Cytopathic effect was monitored daily. Cell supernatants or lysates were collected and subjected to quantitative PCR (qPCR) for MPXV, which was performed as previously described (15). Aliquots were stored at −80°C. For experimental use, frozen stocks were thawed, clarified by centrifugation (3,000 × *g*, 10 min), and the supernatant containing infectious particles was retained for subsequent studies.

Plaque assays were used to quantify the infectious virus titers. Vero E6 cells were seeded into 96-well plates overnight. Tenfold serial dilutions of the virus were prepared in MEM medium containing 2% FBS and 1% antibiotics. Then, 100 µL of each dilution was used to infect Vero E6 cell monolayers. After 72–96 h, cells were fixed with 4% paraformaldehyde (PFA) and stained with crystal violet. Viral titers, expressed as plaque-forming units per milliliter (PFU/mL), were calculated by multiplying the number of plaques by the corresponding dilution factor.

Six-month-old male dormice were housed in ventilated ABSL-3 cages with corn cob bedding and environmental enrichment. Animals were maintained on standard rodent chow (Teklad Global 18% Protein Diet) with *ad libitum* water access following a 7-day acclimatization period.

### Experimental design and drug administration

This study utilized 6-month-old Spanish dormice (*Dryomys nitedula*, *Rodentia*, *Myoxidae*, and *Dryomys*; *n* = 15), as this strain and age are well established in the literature as a model for MPXV infection, exhibiting a predictable disease progression that is optimal for therapeutic evaluation (12). This species exhibits several traits akin to laboratory mice, facilitating its multiplication and maintenance in a research environment, and has been previously validated for evaluating anti-MPXV agents *in vivo* (15), supporting its relevance for the current investigation. Furthermore, the use of male mice in this study eliminates potential confounding effects of hormonal fluctuations on experimental outcomes (16), thereby enhancing the reliability and interpretability of the results.

Six-month-old male dormice with a body weight of 32 ± 3 g were assigned a unique identifier based on body weight and randomly allocated into three groups using a computer-generated randomization sequence (*GraphPad Prism, v9.0*): Group 1: Mock infection (*n* = 5); Group 2: MPXV infection (*n* = 5); Group 3: Aminopterin treatment (*n* = 5).

Dormice were anesthetized via intraperitoneal injection of tribromoethanol (200 mg/kg; Aladdin, Cat#: T161626). Next, they were infected with MPXV (1 × 10^5^ PFU) or DMEM (Mock group) on day 1 by intranasal inoculation. According to a previous study, administration of aminopterin (3–7 mg/kg) daily via intraperitoneal injection in dormice achieved adequate tissue distribution (17, 18). Therefore, for an antiviral study, we selected a lower concentration (1.5 mg/kg) for investigation. Aminopterin (1.5 mg/kg) or an equal volume of vehicle (DMSO-corn oil mixture, 100 μL) was administered via intraperitoneal injection daily from days 2 to 6. Animals were euthanized on day 8, and organs were collected for analysis after the animals were anesthetized. The corn oil vehicle was selected based on a previous study (11).

### Histopathological evaluation

Dormice were euthanized using diethyl ether asphyxiation. Spleens and lungs were perfused with 4% PFA and were then paraffin-embedded. Sections (4 μm thickness) were stained with hematoxylin and eosin. Histopathology was quantified based on a previous study (11). Spleen pathology was graded as follows: Grade 1: Complete red pulp (RP) depletion; Grade 2: Absence of lymphocyte islands with scattered lymphocytes; Grade 3: Markedly reduced lymphocyte island number and size; Grade 4: Slightly reduced lymphocyte island number and size; Grade 5: Normal lymphocyte island morphology.

Lung injury was scored based on alveolar integrity and inflammatory infiltration: no injury (score 0), mild injury (score 1), moderate injury (score 2 or 3), and severe injury (score 4 or 5). Quantitative values were obtained by averaging data from three randomly selected visual fields per animal across five dormice. For histopathological assessment of pulmonary involvement, lung sections from infected dormice were evaluated by two independent pathologists who were blinded to the treatment groups. In cases of scoring discrepancies, a consensus was reached through discussion with a third blinded evaluator. Images were captured using a panoramic tissue imaging system PanoBrain (Meca Scientific, PB-ST100)

### Cytotoxicity assay

A total of 1.0 × 10^4^ cells/well Vero E6 or HFF cells were inoculated in 96-well plates. When the cell confluence reached approximately 90%–100%, different concentrations of aminopterin were added, and the cells were incubated for 48 h. Cell viability was determined using CCK-8 reagent with absorbance measured at 450 nm (TECAN Infinite M200). Results were normalized to untreated controls.

### Viral inhibition assay

Cells were infected with MPXV (MOI = 1) for 2 h, and then they were washed with DMEM and treated with aminopterin for 48 h. Total DNA was collected for further analysis. The half-maximal inhibitory concentration (IC_50_) and cytotoxic concentration (CC_50_) of aminopterin were determined by MPXV-F3L DNA expression levels using real-time qPCR. The selectivity index (SI) for aminopterin was calculated with the formula: SI = CC_50_/IC_50_.

### DNA isolation and qPCR

DNA was extracted using the Universal Genomic DNA Kit. qPCR was performed on an ABI 7500 real-time PCR machine (United States), with 20 μL reaction mixtures containing 2 μL DNA, 10 μL 2× Universal SYBR Green Fast qPCR Mix, 2 μL primers, and 6 μL ddH_2_O. All primers were synthesized by Shenggong Biotech Co., Ltd (Shanghai, China). The primer sequences of MPXV-F3L were as follows: forward primer, 5′-CATCTATTATAGCATCAGCATCAGA-3′, and reverse primer, 5′-GAT ACTCCTCCTCGTTGGTCTAC-3′; the primer sequences of GAPDH were as follows: forward primer, 5′-GAAGGTGAAGGTCGGAGTC-3′, and reverse primer, 5′-GAAGATGGTGATGGGATTTCC-3′. The qPCR reactions containing SYBR Green master mix and gene-specific primers were performed under the following conditions: 95°C for 5 min; 40 cycles of 95°C for 10 s and 60°C for 30 s.

### Time of drug addition assay

The time-of-drug addition assay was performed as previously reported (19, 20). For the −2 h time point, Vero E6 cells were pre-treated with aminopterin (50 nM) for 2 h, followed by infection with MPXV (1 × 10^4^ PFU) for 48 h. For the 0 h time point, Vero E6 cells were simultaneously exposed to MPXV and aminopterin and co-cultured at 37°C. For time points from 2 to 24 h, Vero E6 cells infected with MPXV were treated with aminopterin at 2, 6, 12, and 24 h post-infection, respectively. Viral DNA was quantified at 48 h post-infection to determine the viral load.

### Viral attachment, entry, and replication assay

For the viral attachment assay, Vero E6 cells were co-administered with 100 μL aminopterin (50 nM) and MPXV (1 × 10^4^ PFU), and then they were incubated at 4°C for 1 h. Subsequently, unattached virus-drug mixtures were removed by triple 1 × PBS (pH 7.2) washing. Next, cells were cultured in DMEM without aminopterin and virus for 48 h at 37°C with 5% CO_2_. Finally, intracellular MPXV-F3L DNA levels were used to determine the levels of viral attachment by qPCR assay.

For the viral entry assay, Vero E6 cells were infected with MPXV (1 × 10^4^ PFU) at 4°C for 1 h, and then the unattached viruses were removed by triple 1 × PBS (pH 7.2) washing. Subsequently, they were treated with aminopterin (50 nM) or DMSO vehicle at 37°C for 1 h to assess viral entry. Finally, intracellular MPXV-F3L DNA levels were used to quantify the levels of viral entry by qPCR assay.

For the viral replication assay, Vero E6 cells were infected with MPXV (1 × 10^4^ PFU) for 2 h at 37°C to allow viral attachment and entry. Subsequently, supernatants were then discarded, and cells were washed three times with 1 × PBS (pH 7.2). Next, fresh DMEM with aminopterin (50 nM) was added to the plates and cultured at 37°C for 46 h. Finally, the viral DNA was used to determine the levels of the replication of the virus by qPCR assay.

### Enzyme-linked immunosorbent assay

Interleukin-1 beta (IL-1β), IL-6, tumor necrosis factor-alpha (TNF-α), and interferon-gamma (IFN-γ) levels in the supernatant of HFF cells were detected by enzyme-linked immunosorbent assay (ELISA) kits, which were purchased from Lianke Biotechnology Co., Ltd. (Cat#: EK101B-48T, EK106-48T, EK182-48T, and EK180-48T; Hangzhou, China). The experimental operation was carried out strictly according to the instructions.

### Statistical analysis

qPCR data were analyzed using the 2^−△△CT^ method with normalization to the housekeeping gene *gapdh*. One-way analysis of variance , SNK-q test, and two-tailed Student’s *t-*test, using PRISM software (version 9; GraphPad software). The *P* values < 0.05 were considered statistically significant.

## RESULTS

### Aminopterin exhibits anti-MPXV effects *in vitro*

**Fig 1:**
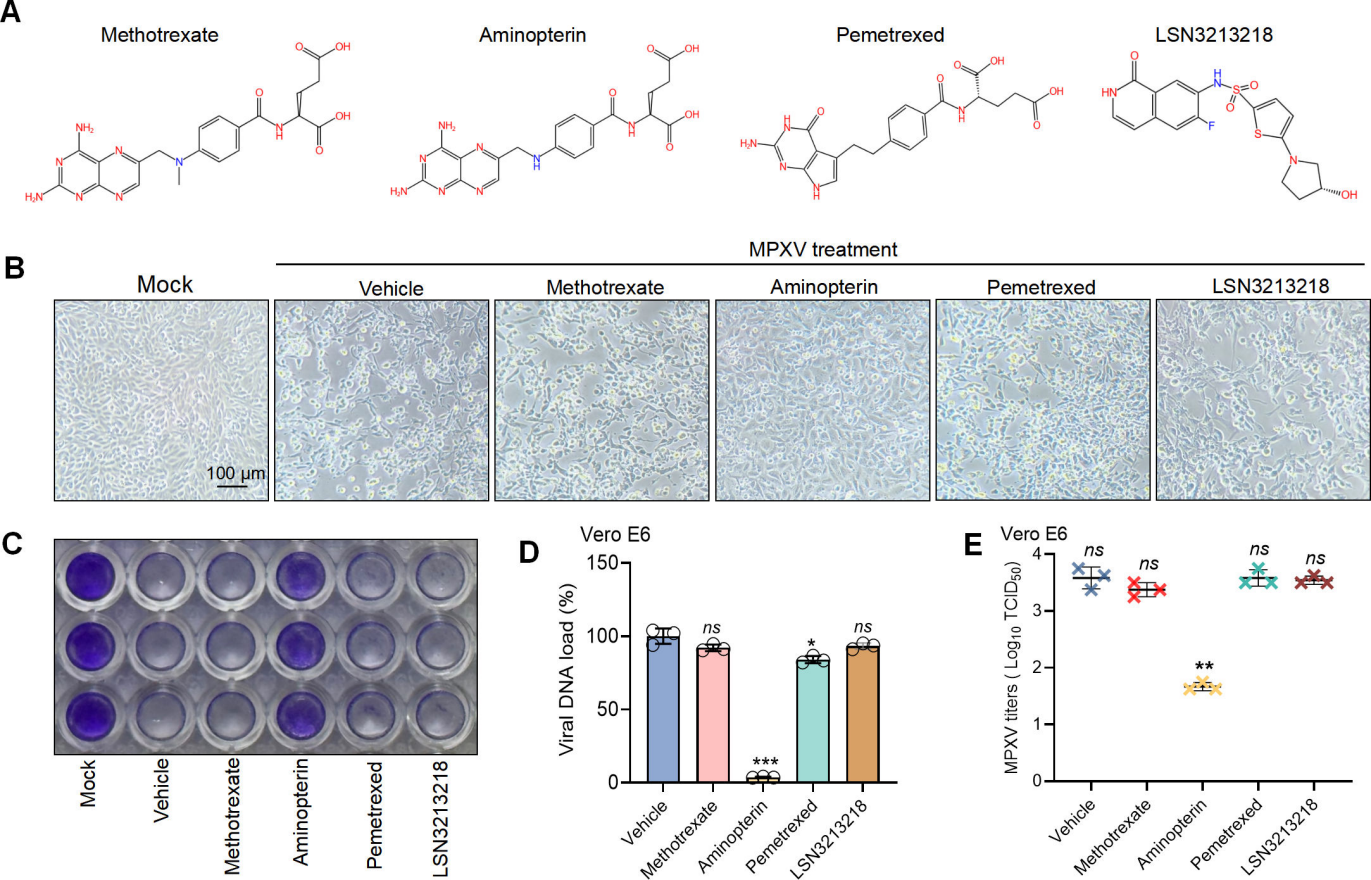
Screening of anti-MPXV infection among the folate antagonists. (**A**) Chemical structures of MTX, aminopterin, pemetrexed, or LSN3213218 are shown. (**B and C**) Vero E6 cells were treated with MTX, aminopterin, pemetrexed, or LSN3213218 at 100 nM following MPXV (1 × 10^4^ PFU) infection for 48 h. Morphology changes in MPXV-infected cells following the drug treatment were assessed by light microscopy (scale bar: 100 μm), followed by crystal violet staining to quantify cytoprotection. (**D**) Viral DNA load reduction rate of MPXV infection by MTX, aminopterin, pemetrexed, or LSN3213218 was calculated via qPCR analysis of intracellular viral DNA (*n* = 3, independent experiments). (**E**) Viral titers in cell supernatants from panels **B** and **C** were determined by TCID_50_ assay (*n* = 3, independent experiments). Data expressed as means of technical replicates ± SD, and one-way analysis of variance (**D and E**) and SNK-q test (**D and E**) were performed in this section (*P < 0.05, **P < 0.01, and ***P < 0.001, ns: not significant).

Folate is a crucial nutrient that supports various physiological functions (21). Our prior study identified a folate antagonist with antiviral effects both *in vitro* and *in vivo* (11, 22). Here, we screened several antifolate agents, including MTX, aminopterin, pemetrexed, and LSN3213218, for anti-MPXV activity via a cell-based infection assay. Among these, only aminopterin (4-Aminofolate), the 4-amino derivative of folate, significantly inhibited MPXV replication in infected Vero E6 cells (Fig. 1B). Crystal violet staining confirmed the cytoprotective effect of aminopterin treatment (Fig. 1C). In validation assays under identical conditions, a marked reduction in intracellular MPXV DNA levels was observed after treatment with 100 nM aminopterin for 48 h (Fig. 1D). Consistently, 100 nM aminopterin also significantly suppressed the production of MPXV virions (Fig. 1E). Collectively, these findings establish aminopterin as a potent inhibitor of MPXV *in vitro*.

### Dose-dependent anti-MPXV activity of aminopterin

**Fig 2:**
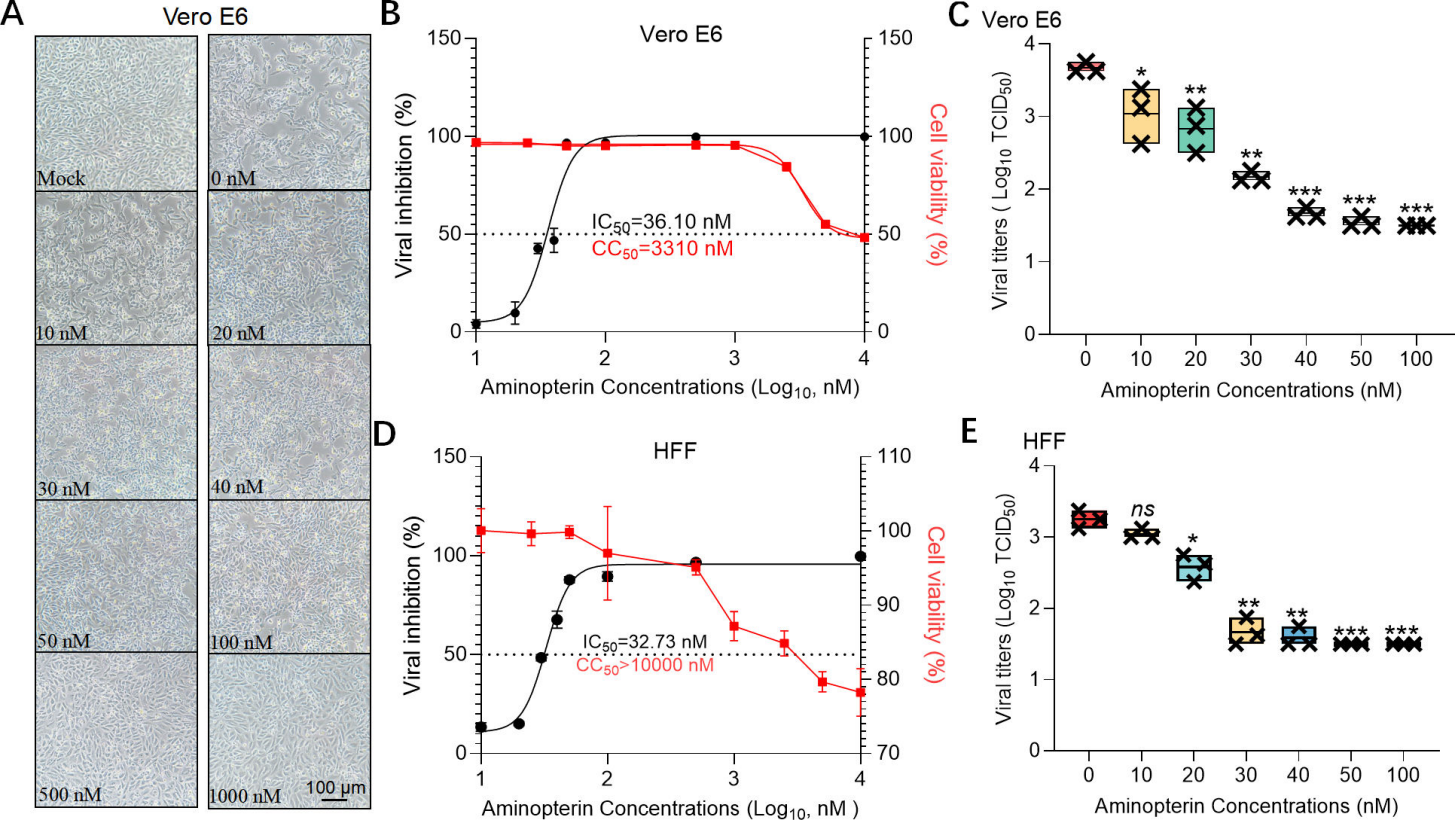
Aminopterin potently inhibits MPXV replication *in vitro*. (**A**) Morphological analysis of MPXV-infected Vero E6 cells (1 × 10^4^ PFU) treated with aminopterin (0–1 μM, 48 h) by light microscopy (scale bar: 100 μm). (**B and D**) CC_50_ and IC_50_ of aminopterin were calculated by linear regression analysis of the viral DNA inhibition curves in Vero E6 and HFF cells, using qPCR assay. SI value was defined as the ratio of CC_50_ to IC_50_ (CC_50_/IC_50_; *n* = 3, independent experiments). (**C and E**) Infectious viral titers in supernatants from panels **B** and **D** were determined by TCID_50_ assay (*n* = 3, independent experiments). Data expressed as means of technical replicates ± SD, and one-way analysis of variance (**C and E**) and SNK-q test (**C and E**) were performed in this section (*P < 0.05, **P < 0.01, and ***P < 0.001**,** ns: not significant).

Aminopterin inhibited MPXV infection in Vero E6 cells in a dose-dependent manner (0–1,000 nM) for 48 h (Fig. 2A). The IC_50_ and CC_50_ of aminopterin were 36.10 nM and 3,310 nM in Vero E6 cells and 32.73 nM and >10,000 nM in HFF cells (Fig. 2B and D). The corresponding SI value of aminopterin was 91.690 for Vero E6 cells and >305.530 for HFF cells. Consistent with these findings, viral titers decreased dose dependently in both cell lines (Fig. 2C and E).

### Aminopterin interrupts MPXV replication at the early stage of viral infection

**Fig 3:**
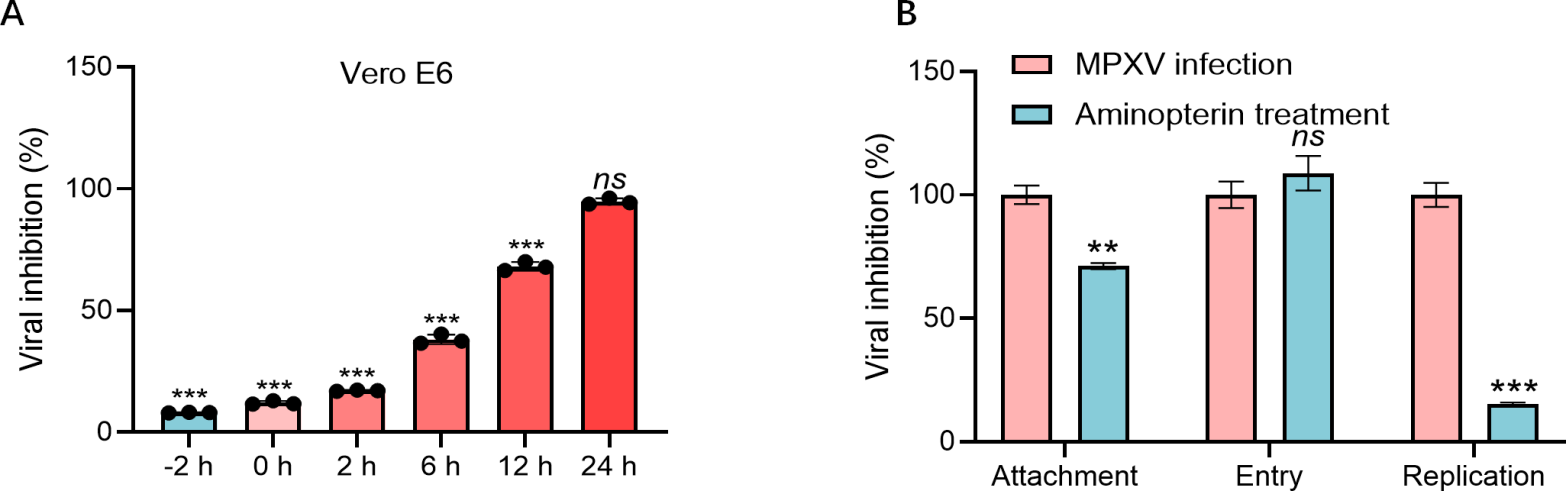
Aminopterin interrupts viral attachment and replication. (**A**) Vero E6 cells were treated with aminopterin at various time points (−2 h, 0 h, 2 h, 6 h, 12 h, and 24 h). Furthermore, Vero E6 cells were infected with MPXV (1 × 10^4^ PFU) for 48 h, with viral inoculation performed at 0 h in the presence of aminopterin. Intracellular MPXV-F3L DNA content was determined by qPCR assay (*n* = 3, independent experiments). (**B**) Intracellular MPXV-F3L DNA levels were determined by qPCR analysis in the attachment assay, entry assay, and replication assay (*n* = 3, independent experiments). Data expressed as means of technical replicates ± SD and Student’s *t*-test (**A and B**) were performed in this section (**P < 0.01, and ***P < 0.001, ns: not significant).

Based on the finding that aminopterin exhibited an IC_50_ of 36.1 nM against MPXV in Vero E6 cells and approached optimal antiviral activity at 50 nM without cytotoxicity (Fig. 2), this concentration was selected for subsequent *in vitro* experiments. To identify the specific phase of the MPXV life cycle inhibited by aminopterin, we performed a time-of-addition assay. The results showed that aminopterin primarily inhibited MPXV replication at the early stage of viral infection (Fig. 3A). Furthermore, aminopterin treatment also inhibited viral attachment by approximately 20% and viral replication by approximately 70% but had a minimal effect on viral entry (Fig. 3B). These findings are based on results of entry and replication assays showing that aminopterin interrupts MPXV replication at the early stage of viral infection.

### Aminopterin mitigates MPXV pathogenesis *in vivo*

**Fig 4:**
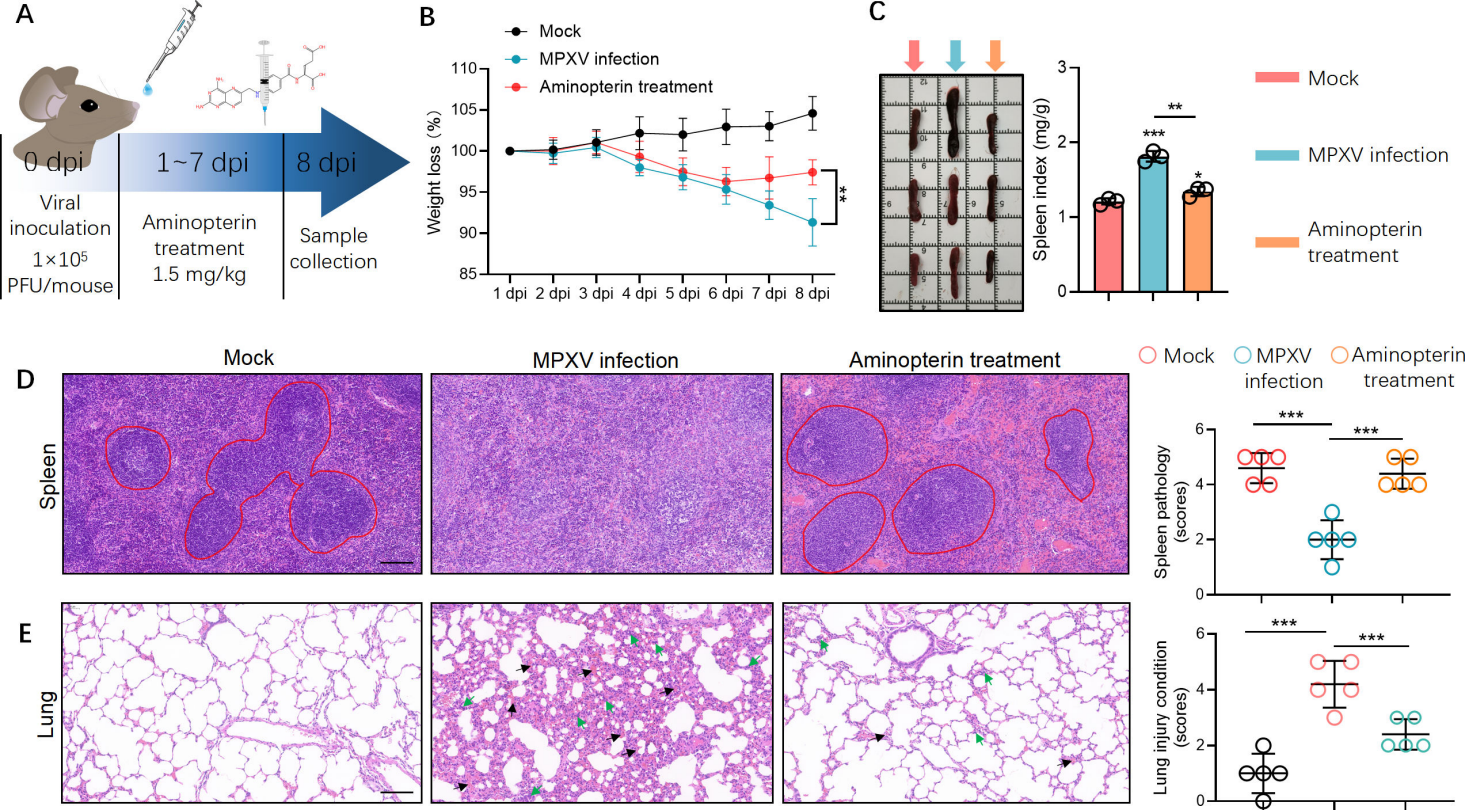
Aminopterin alleviates spleen and lung injury after MPXV infection *in vivo*. (**A**) Schematic of the animal experiment design. Dormice were anesthetized and infected intranasally with MPXV (total 1 × 10^5^ PFU) on day 1. Aminopterin (1.5 mg/kg) or vehicle was administered via intraperitoneal injection daily from days 2 to 8. Subsequently, dormice were euthanized on day 8, and the organs were collected for analysis (*n* = 5, animals, biological replicates). (**B**) The percent of body weight was monitored for 8 days among the dormice challenged with MPXV (1 × 10^5^ PFU) and treated with aminopterin and vehicle, respectively (*n* = 5, animals, biological replicates). (**C**) Splenic index was calculated with spleen-to-body weight ratio (mg/g; *n* = 3, animals, biological replicates). (**D and E**) Histopathology changes in lung and spleen tissues, respectively (scale bar: 100 μm). Quantification standards are detailed in the Materials and Methods section (*n* = 5, animals, biological replicates). Red lines indicate the RP area. Green arrows indicate lymphocytic infiltration. Black arrows indicate interstitial hemorrhage. Data were expressed as means of technical replicates ± SD. One-way analysis of variance (**C, D, and E**) and SNK-q test (**C, D, and E**) were performed in this section (*P < 0.05, **P < 0.01, and ***P < 0.001, *ns***:** not significant).

To evaluate the therapeutic potential of aminopterin against MPXV/SZTH42 infection, we conducted an *in vivo* study using a dormouse model. Dormice were intranasally inoculated with 1 × 10^5^ PFU of MPXV/SZTH42 on day 1. The infected dormice were then treated intraperitoneally with either aminopterin (1.5 mg/kg) or vehicle from days 2 to 6. All animals were euthanized on day 8 for endpoint analysis, and the indicated time points tissues were collected for further analysis (Fig. 4A). MPXV-infected animals exhibited lethargy, ruffled fur, and reduced activity throughout the infection, whereas aminopterin treatment led to an improvement in these signs in 40% (2/5) of the dormice starting on 5 days post-infection (dpi), with the improvement rate reaching 80% (4/5) by 8 dpi. Infected control animals exhibited progressive weight loss from 3 to 7 dpi, whereas aminopterin-treated animals began to regain weight by 6 dpi (Fig. 4B). Histopathological evaluation revealed significant pathology in the infected controls. The MPXV-infected group developed marked splenomegaly, as indicated by increased spleen indices compared to the mock-infected controls. Aminopterin treatment effectively mitigated this splenic enlargement (Fig. 4C). Splenic histology further demonstrated that aminopterin administration restored lymphocyte populations in the RP, as delineated by the red lines (Fig. 4D). Lung tissue analysis revealed that MPXV infection induced severe pulmonary pathology, characterized by diminished alveolar spaces, lymphocytic infiltration (green arrows), and interstitial hemorrhage (black arrows). In contrast, aminopterin-treated dormice maintained near-normal lung architecture, exhibiting only mild hemorrhage and alveolar lymphocytic infiltration (Fig. 4E). Collectively, these findings indicate that aminopterin treatment promotes rapid weight recovery, ameliorates MPXV-associated splenic pathology, and attenuates pulmonary tissue damage in MPXV-infected dormice.

### Aminopterin inhibits systemic MPXV replication and reduces virus-associated inflammatory factors levels *in vivo*

**Fig 5:**
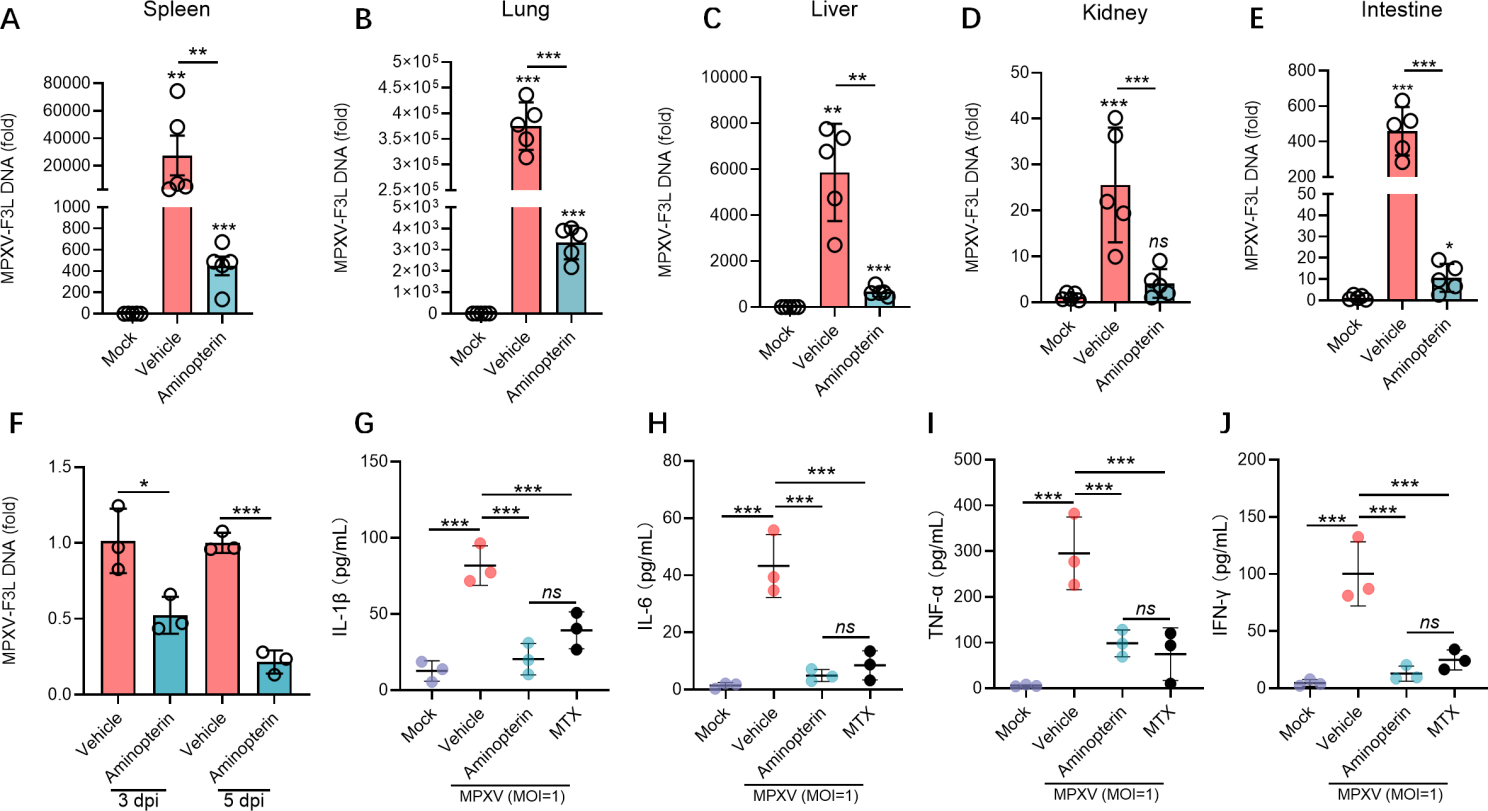
Aminopterin inhibits systemic MPXV replication and reduces virus-associated inflammatory factors levels *in vivo.* (**A–E**) The levels of MPXV-F3L DNA in lung, spleen, liver, kidney, and intestine tissues at 8 dpi were detected by qPCR (*n* = 5, animals, biological replicates). (**F**) The levels of MPXV-F3L DNA in lung tissues at 3 dpi and 5 dpi were detected by qPCR (*n* = 3, animals, biological replicates). (**G–J**) The levels of IL-1β, IL-6, TNF-α, and IFN-γ in the supernatant of MPXV-infected HFF cells were detected by ELISA assay (*n* = 3, biological replicates). Data were expressed as means of technical replicates ± SD. One-way analysis of variance (**A–I**) and SNK-q test (**A–I**) were performed in this section (*P < 0.05, **P < 0.01, and ***P < 0.001, *ns***:** not significant).

Studies of liver, lung, spleen, kidney, and intestine are most informative for poxvirus dissemination and transmission (15). We subsequently quantified MPXV-F3L DNA levels in the spleen, lungs, liver, intestine, and kidney tissues of dormice. As shown in Fig. 5A through E, aminopterin-treated dormice exhibited lower viral DNA levels across these organs compared to the untreated MPXV-infected group. Subsequently, we evaluated the antiviral activity of aminopterin during the early stages of viral infection *in vivo*. Aminopterin significantly decreased the expression of the MPXV-F3L protein in lung tissues at both 3 and 5 dpi (Fig. 5F). Since a systemic inflammatory storm triggered by viral infection is a key contributor to tissue damage (23, 24), and given that multiple inflammatory factors, including IL-1β, IL-6, TNF-α, and IFN-γ, are known to be elevated during MPXV and other viral infections (25–27). We next investigated the anti-inflammatory effects of aminopterin during MPXV infection. MTX is an analog of aminopterin and a well-known anti-inflammatory agent (28), served as a parallel control without anti-MPXV activity in this study. The results showed that both aminopterin and MTX treatment significantly reduced the levels of IL-1β, IL-6, TNF-α, and IFN-γ in MPXV-infected HFF cells supernatant (Fig. 5G through J). These data indicate that aminopterin not only inhibits MPXV replication in multiple organs but also reduces virus-associated systemic inflammation *in vivo*.

## DISCUSSION

The development of MPXV-specific antiviral agents is critical for the effective management of Mpox infections (29). Although antifolate agents were originally developed as anticancer drugs, emerging evidence highlights their broader pharmacological potential (30, 31). Our study provides the first evidence that the classic antifolate compound aminopterin exhibits potent anti-MPXV activity in both *in vitro* and *in vivo* models.

Historically, aminopterin induced the first remission in pediatric acute lymphoblastic leukemia (ALL), representing a milestone in cancer chemotherapy (9, 32). It has also been used as a treatment for severe psoriasis (33). Recent work by our group identified the antiviral activity of antifolate agents such as MTX and pralatrexate (11, 34); however, the activity of this class against MPXV remained largely unknown. Our systematic screening of four antifolates (MTX, aminopterin, pemetrexed, and LSN3213218) identified aminopterin as the only compound that significantly inhibited MPXV replication across experimental models. In Vero E6 cells, aminopterin exhibited an SI value of 91.69 against MPXV. Tecovirimat has been approved for Mpox in Europe and is available in the United States via an expanded-access investigational new drug protocol. However, its clinical efficacy remains debated (35). For example, tecovirimat shows limited effectiveness against Clade I MPXV strains (36). One study reported that tecovirimat has a low IC_50_ (12.73) and a high SI value (>7,855) for MPXV in Vero E6 (15), which may appear advantageous compared to aminopterin in this specific context. It should be noted that SI values are highly dependent on detection conditions and cell types and cannot alone reflect selective target of pathogens; therefore, the results of this study should be interpreted with caution.

Although several antivirals, including tecovirimat, brincidofovir, cidofovir, NIOCH-14, and trifluridine, have shown activity against orthopoxviruses, clinical options remain limited (37). Notably, tecovirimat recently did not meet endpoints in Phase III trials (RCTs PALM007 and STOMP), potentially due to delayed treatment, suboptimal drug exposure, or emerging resistance (36). These limitations underscore the need for new therapeutic strategies. Aminopterin may represent a distinct mechanism of action, differing from direct-acting antivirals by targeting host cell pathways essential for viral replication rather than viral components themselves. Its potential could be explored as monotherapy for resistant infections or in combination regimens to enhance efficacy and mitigate resistance. However, our conclusions are constrained by the use of a single viral strain and animal model. Future studies should include diverse strains and more relevant models to better define its broad-spectrum potential and therapeutic applicability.

In this study, we demonstrate that aminopterin significantly inhibits MPXV replication and attachment *in vitro*. The antiviral activity has been attributed to its dual targeting of host and viral components. First, aminopterin competitively antagonizes folate metabolism by inhibiting DHFR, a critical enzyme for nucleotide synthesis (9). DHFR inhibition decreases intracellular deoxyribonucleotide pools of dTTP, dATP, and dGTP (38), which may block viral DNA replication and further reduce progeny viral production (39, 40). However, in this study, the lack of antiviral effect from other DHFR-targeting antifolates implies a pathway distinct from classical folate metabolism inhibition. This observation aligns with our recent findings demonstrating limited reliance of DNA viruses on host folate pathways compared to RNA viruses (41). Furthermore, another antifolate, pralatrexate, inhibits the MPXV replication independently of its antifolate activity, but rather by upregulating the expression of antiviral proteins APOBEC3s (15). Together, these results strongly suggest that aminopterin exerts its anti-MPXV effects primarily through non-canonical mechanisms that warrant further investigation. Second, aminopterin pretreatment exhibits approximately 20% inhibition of MPXV adsorption to host cells in this study. Glycosaminoglycan (GAG)-mediated cell surface interaction (42, 43) represents a primary mechanism by which the MPXV utilizes heparan sulfate (HS)-dependent pathways for cellular entry (44). However, direct evidence that aminopterin disrupts MPXV binding to GAGs or HS is currently lacking. Furthermore, we speculate that this phenomenon may be attributed to the intrinsic molecular properties of aminopterin. Under the experimental conditions (co-treatment of cells with aminopterin and MPXV for 1 h at 4°C, which primarily permits viral adsorption while inhibiting internalization), there may have been sufficient time for aminopterin to interact with viral surface proteins, thereby interfering with the virus’s ability to adsorb to host cells. This hypothesis is supported by previous findings demonstrating aminopterin’s high binding affinity for viral proteins to limit SARS-CoV-2 entry (45). Nevertheless, in the absence of direct mechanistic evidence, these results should be interpreted with caution.

Despite its efficacy in early clinical trials for acute ALL, aminopterin was largely superseded by MTX in the 1950s due to the latter’s more favorable therapeutic index (46). This transition limited the exploration of aminopterin’s potential in other therapeutic areas where its distinct biochemical properties might offer unique advantages. Previous studies have reported that aminopterin exhibits greater potency and bioavailability than MTX (47, 48). It also demonstrates lower accumulation in the central nervous system, suggesting a potentially improved neurotoxicity profile (47). The toxicity of antifolates primarily manifests as symptoms of folate deficiency, such as oral mucositis, bone marrow suppression, and gastrointestinal disturbances (49). These adverse effects are typically associated with long-term, high-dose administration and can often be reversed by high-dose leucovorin or folate supplementation (50). MPXV infection generally requires a shorter treatment duration than malignancies, potentially allowing for brief courses of aminopterin. Nevertheless, aminopterin has been classified as an extremely hazardous substance in the United States (51), and the daily dose of 1.5 mg/kg used in this study remains relatively high and may still pose safety concerns. For instance, despite suppressing ~50% of lung viral load by 3 dpi, aminopterin treatment led to only partial body weight recovery by 8 dpi, indicating potential safety concerns that merit cautious interpretation. Future studies could explore whether lower or optimized dosing regimens, when tested in MPXV models, maintain efficacy while improving tolerability. Alternatively, molecular modification of aminopterin to reduce its toxicity represents another viable strategy. If folate supplementation can effectively mitigate aminopterin-related toxicity, as suggested previously (50), such mitigation would indicate that its adverse effects might be manageable in the context of MPXV therapy. However, given that our prior work suggests that folate supplementation could potentially exacerbate RNA viral infections (34, 41), this approach requires careful evaluation and further investigation.

In the dormice model, MPXV infection induced severe pulmonary pathology, characterized by diminished alveolar spaces, lymphocytic infiltration, and interstitial hemorrhage. In contrast, aminopterin-treated dormice maintained near-normal lung architecture, exhibiting only mild hemorrhage and alveolar lymphocytic infiltration (Fig. 4E). Aminopterin treatment alleviated MPXV-induced lung pathology and reduced key pro-inflammatory cytokine levels. Aminopterin reduced the levels of cytokines such as IL-1β, IL-6, TNF-α, and IFN-γ in the supernatant of MPXV-infected HFF cells. Consistent with a previous study that identified aminopterin’s anti-inflammatory properties in rheumatoid arthritis models (52), our results suggest that the drug has the capacity to concurrently mitigate viral propagation and hyper-inflammation driven by MPXV infection. Interestingly, MTX can also decrease the levels of these cytokines with no anti-MPXV properties in this study, which imply that MTX may share a similar anti-inflammatory profile with aminopterin but not anti-MPXV activity. Future work should thus incorporate lung tissue-based assessments, such as inflammatory gene expression and immune cell profiling, to more comprehensively evaluate the anti-inflammatory activity of aminopterin.

The Spanish dormouse (*Dryomys nitedula*) was selected as the primary *in vivo* model for this study due to its well-documented susceptibility to both Clade I and Clade II MPXV strains, a feature lacking in conventional murine models (including C57BL/6 and BALB/c) (53). Unlike CAST/EiJ mice, which show no clinical signs of disease following Clade IIb MPXV challenge, dormice develop robust infection phenotypes, including significant weight loss and multi-organ pathology, enabling reliable evaluation of antiviral efficacy (54). While non-human primates and other larger models offer greater immunological relevance, the dormouse model provides a pragmatic balance between physiological relevance, as a natural MPXV reservoir species, and practical advantages, such as compact size, reduced biosafety containment requirements, and cost-effectiveness, which collectively ensure reproducible disease progression (55, 56).

However, our study has some limitations. The generalizability of the findings is constrained by the use of a single MPXV strain and animal model. Furthermore, the evaluation was conducted under a single aminopterin dose and treatment schedule, and the absence of comprehensive immune profiling and detailed pharmacokinetic data limits the current understanding of its therapeutic profile. Future investigations incorporating multiple viral strains, optimized dosing regimens, and extended immunologic analyses. Furthermore, the toxicity of aminopterin is well recognized in the United States, which might limit its usage in clinical practice. Therefore, further study has to consider pharmacokinetic-toxicological evaluations to fully establish the translational relevance of aminopterin as an anti-MPXV agent. Another limitation is the lack of mechanistic evidence on how aminopterin inhibits MPXV attachment and replication, and its anti-inflammatory mechanism remains unclear. These results should be interpreted with caution. Thus, these limitations must be considered when attempting to translate our findings into a clinical context.

In summary, aminopterin emerges as a dual-action therapeutic candidate against MPXV, combining viral replication inhibition with inflammation modulation. This discovery provides a critical foundation for developing countermeasures against the escalating global threat of Mpox, offering new strategies to combat this re-emerging infectious disease.
